# Design of Mid‐Q Response: A prospective, randomized trial of adaptive cardiac resynchronization therapy in Asian patients

**DOI:** 10.1002/joa3.12731

**Published:** 2022-05-20

**Authors:** Kengo Kusano, Seung‐Jung Park, Sofian Johar, Toon Wei Lim, Bart Gerritse, Kazuhiro Hidaka, Kazutaka Aonuma

**Affiliations:** ^1^ Department of Cardiovascular Medicine National Cerebral and Cardiovascular Center Osaka Japan; ^2^ Sungkyunkwan University School of Medicine Samsung Medical Center Seoul South Korea; ^3^ Gleneagles Jerudong Park Medical Centre and Institute of Health Sciences Universiti Brunei Darussalam Bandar Seri Begawan Brunei Darussalam; ^4^ National University Hospital Singapore Singapore; ^5^ Medtronic Bakken Research Center Maastricht The Netherlands; ^6^ Medtronic Japan Tokyo Japan; ^7^ Department of Cardiology, Faculty of Medicine University of Tsukuba Tsukuba Japan

**Keywords:** adaptive CRT, atrioventricular conduction, cardiac resynchronization therapy, clinical outcome, left bundle branch block, left ventricular pacing

## Abstract

**Aims:**

The aim of the Mid‐Q Response study is to test the hypothesis that adaptive preferential left ventricular‐only pacing with the AdaptivCRT algorithm has superior clinical outcomes compared to conventional cardiac resynchronization therapy (CRT) in heart failure (HF) patients with moderately wide QRS duration (≥120 ms and <150 ms), left bundle branch block (LBBB), and normal atrioventricular (AV) conduction (PR interval ≤200 ms).

**Methods:**

This prospective, multi‐center, randomized, controlled, clinical study is being conducted at approximately 60 centers in Asia. Following enrollment and baseline assessment, eligible patients are implanted with a CRT system equipped with the AdaptivCRT algorithm and are randomly assigned in a 1:1 ratio to have AdaptivCRT ON (Adaptive Bi‐V and LV pacing) or AdaptivCRT OFF (Nonadaptive CRT). A minimum of 220 randomized patients are required for analysis of the primary endpoint, clinical composite score (CCS) at 6 months post‐implant. The secondary and ancillary endpoints are all‐cause and cardiovascular death, hospitalizations for worsening HF, New York Heart Association (NYHA) class, Kansas City Cardiomyopathy Questionnaire (KCCQ), atrial fibrillation (AF), and cardiovascular adverse events at 6 or 12 months.

**Conclusion:**

The Mid‐Q Response study is expected to provide additional evidence on the incremental benefit of the AdaptivCRT algorithm among Asian HF patients with normal AV conduction, moderately wide QRS, and LBBB undergoing CRT implant.

## 1 INTRODUCTION

Cardiac resynchronization therapy (CRT) is an established therapy that has been shown to reduce morbidity and mortality in select symptomatic heart failure (HF) patients with left ventricular systolic dysfunction.^1^, ^2^ However, a lack of overt clinical improvement has been reported in up to one‐third of indicated patients.^3^ Patient‐specific characteristics, such as severity and type of electrical conduction abnormalities, dyssynchrony, and scar burden, have been reported to affect the degree of CRT response.^4^, ^5^ Additionally, device factors, such as suboptimal atrioventricular (AV) timing and suboptimal lead position also contribute to the sub‐optimal response.^3^

While CRT is most commonly achieved through biventricular (BiV) pacing, previous studies have demonstrated that left ventricular (LV) only pacing can be equally as effective as BiV pacing.^5^, ^6^ In patients with sinus rhythm and normal AV conduction, pacing only the LV with appropriate AV delays can result in superior LV^5^, ^7^ and right ventricular (RV)^8^, ^9^ function compared to BiV pacing.

The AdaptivCRT algorithm adjusts AV and VV delays based on periodic automatic evaluation of intrinsic conduction intervals. When intrinsic AV conduction time is normal, the algorithm provides RV‐synchronized LV pacing, and BiV pacing when AV conduction is significantly delayed or blocked. The Adaptive CRT study demonstrated non‐inferiority of AdaptivCRT on the Clinical Composite Score (CCS) compared to echo‐optimized BiV pacing.^10^ In a subsequent subgroup analysis among patients with normal AV conduction, left bundle branch block (LBBB), and moderately wide QRS (120–150 ms), a significant improvement in CCS was observed in the AdaptivCRT arm compared to the echo‐optimized arm.^11^

Notably, U.S. and European guidelines, which are supported by the initial evidence of CRT effectiveness demonstrated primarily in Western countries with Caucasian populations, provide the strongest level of recommendation (Class I) for patients with a QRS duration of ≥150 ms in conjunction with LBBB and reduced left ventricular ejection fraction (LVEF).^1^, ^2^ However, QRS duration tends to vary by height, heart size, and race. On average, narrower QRS complexes are more frequently observed in Asian adults compared to Caucasian adults, which is often attributed to smaller heart sizes in Asians.^12^ In addition, Asian HF patients also tend to have more severe impairment in LVEFs.

Taken together, we hypothesized that Asian HF patients with QRS durations at the lower end of the spectrum could benefit from AdaptivCRT. The Mid‐Q Response study was designed as a prospective, multicenter, randomized controlled trial to test the hypothesis that preferential LV‐only pacing with the AdaptivCRT algorithm is superior to conventional BiV pacing in regards to patient outcomes among Asian HF patients with moderately prolonged QRS duration (≥120 ms and <150 ms), LBBB, and normal AV conduction (PR interval ≤200 ms).

## 2 STUDY DESIGN

Mid‐Q Response is a prospective, randomized, parallel‐arm, single‐blinded, multi‐center study in CRT indicated patients. The study is being conducted at approximately 60 centers in Asia, including centers in Japan, China, Taiwan, South Korea, Hong Kong, Malaysia, Singapore, Indonesia, Brunei, and the Philippines. Up to 232 patients will be enrolled in order to include the 220 randomized patients needed for the primary endpoint analysis. The first patient was enrolled in January 2020 and enrollment completion is expected to take place in approximately January 2023. However, because of the unknown duration and impact of the COVID‐19 pandemic on CRT implants in the participating countries, the enrollment period may be extended beyond this time. Written approval from each center's Institutional Review Board and/or Medical Ethics Committee was obtained, and all patients will provide written informed consent.

Eligible patients are considered enrolled once consent is obtained. In most cases, patients will then undergo the baseline assessment followed by an implant of a market‐approved CRT system containing the AdaptivCRT algorithm. However, patients may also be enrolled within 3 days after implant if all the inclusion criteria and none of the exclusion criteria are met. In these cases, patients' baseline assessments must reflect their pre‐implant status. Within 7 days of a successful implant, the patients will be randomized in a 1:1 fashion to either the treatment (AdaptivCRT ON, Adaptive Bi‐V and LV) or control (AdaptivCRT OFF, conventional BiV pacing) arm. The randomization will be stratified by site, using random permuted blocks of sizes 2 and 4, with blocks in random order. In order to minimize bias, patients will be blinded to their randomization assignment during the participation period.

The ECG Core Laboratory will review all baseline ECGs for the presence of LBBB in addition to QRS duration ≥120 ms and <150 ms and normal AV conduction. Feedback on accuracy rates will be presented to the Steering Committee (see Appendix 1) and to the sites. Additional methods incorporated in the study design to minimize potential bias include: (i) to ensure widespread distribution of data between centers, the maximum number of randomized patients allowed per center is no more than 50, (ii) data collection requirements and study procedures will be standardized across all centers and geographies, (iii) monitoring visits will be conducted for adherence to the protocol and to verify the collected data against the source data, (iv) the Steering Committee members will not have an influence on the HF treatment decisions by center investigators during the trial, except for approval for crossover, and (v) the analysis will be intent‐to‐treat, following pre‐defined statistical methods specified in the protocol and the statistical analysis plan.

### 2.1 Study population and enrollment

The Mid‐Q Response study will include HF patients in Asia indicated for CRT per local guidelines with New York Heart Association (NYHA) class II, III, or IV despite optimal medical therapy, moderately wide QRS (duration ≥120 ms and < 150 ms), preserved AV conduction (PR interval ≤200 ms), and LBBB defined as QS or rS in leads V1 and V2, and mid‐QRS notching or slurring in ≥2 of leads V1, V2, V5, V6, I, and aVL. The definition of LBBB is a modification of the Strauss criteria^13^ with adapted QRS requirements. Optimal medical therapy is defined as a maximally tolerated dose of beta‐blockers and a therapeutic dose of an angiotensin‐converting enzyme inhibitor (ACE‐I), angiotensin receptor blocker (ARB), or mineralocorticoid receptor antagonist (MRA). Patients will be screened to ensure they meet all the Mid‐Q Response inclusion and none of the exclusion criteria prior to study enrollment. A complete overview of the inclusion and exclusion criteria is provided in Table 1.

**TABLE 1:** Study inclusion and exclusion criteria

Inclusion criteria	Exclusion criteria
Patient is willing to sign and date the study consent form. Patient is indicated for a CRT device according to local guidelines. Patient has minimally: Sinus rhythm at time of enrollment. LBBB as documented on an electrocardiogram (ECG) (preferably within 30 days prior to enrollment but up to 50 days is accepted) with moderately wide QRS^a^: Intrinsic QRS duration ≥120 ms and <150 ms QS or rS in leads V1 and V2, and Mid‐QRS notching or slurring in ≥2 of leads V1, V2, V5, V6, I, and aVL. Intrinsic, normal AV conduction as documented on an ECG by a PR interval ≤ 200 ms (preferably within 30 days prior to enrollment but up to 50 days is accepted). LVEF ≤35% (documented within 180 days prior to enrollment). NYHA class II, III or IV (documented within 30 days prior to enrollment) despite optimal medical therapy. Optimal medical therapy is defined as maximal tolerated dose of beta blockers and a therapeutic dose of ACE‐I, ARB or MRA.	Patient is less than 18 years of age (or has not reached minimum age per local law if that is higher). Patient is not expected to remain available for at least 1 year of follow‐up visits. Patient has permanent atrial arrhythmias for which pharmacological therapy and/or cardioversion have been unsuccessful or have not been attempted. Patient is, or previously has been, receiving CRT. Patient is currently enrolled or planning to participate in a potentially confounding drug or device trial during the course of this study. Co‐enrollment in concurrent trials is only allowed when documented pre‐approval is obtained from the study manager. Patient has unstable angina or experienced an acute myocardial infarction or received coronary artery revascularization or coronary angioplasty within 30 days prior to enrollment. Patient has a mechanical tricuspid heart valve or is scheduled to undergo valve repair or valve replacement during the course of the study. Patient is post heart transplant (patients on the heart transplant list for the first time are not excluded). Patient has a limited life expectancy because of non‐cardiac causes that would not allow completion of the study. Patient is pregnant (if required by local law, women of child‐bearing potential must undergo a pregnancy test within seven days prior to device implant). Patient meets any exclusion criteria required by local law.

Abbreviations: ACE‐I, Angiotensin Converting Enzyme Inhibitor; ARB, Angiotensin Receptor Blocker; AV, atrio‐ventricular; CRT, Cardiac Resynchronization Therapy; ECG, electrocardiogram; LBBB, Left Bundle Branch Block; LVEF, Left Ventricular Ejection Fraction; MRA, Mineralocorticoid Receptor Antagonist; NYHA, New York Heart Association.

^a^ This definition of LBBB is based on Strauss et al.^13^ with modification of the QRS duration.

### 2.2 Study conduct

The Mid‐Q Response clinical study will be conducted in compliance with the protocol and federal, national and local laws, regulations, standards, and requirements of the countries/geographies where the study is being conducted. In Japan, the study will be conducted in compliance with the Clinical Trials Act. The principles of the Declaration of Helsinki are implemented by means of the patient informed consent process, Ethics Committee approval, study training, clinical trial registration, risk‐benefit assessment, and publication policy. The Mid‐Q Response clinical study is designed with good clinical practice (GCP) principles as guidance. These include the protection of the rights, safety, and well‐being of human patients, controls to ensure the scientific conduct and credibility of the clinical investigation, and the definition of responsibilities of the sponsor and investigators.

The study was publicly registered prior to first enrollment on http://clinicaltrials.gov (ID: NCT04180696) and the Japan Registry of Clinical Trials (https://jrct.niph.go.jp ID: jRCTs052190113).

### 2.3 Study flow

**FIGURE 1:**
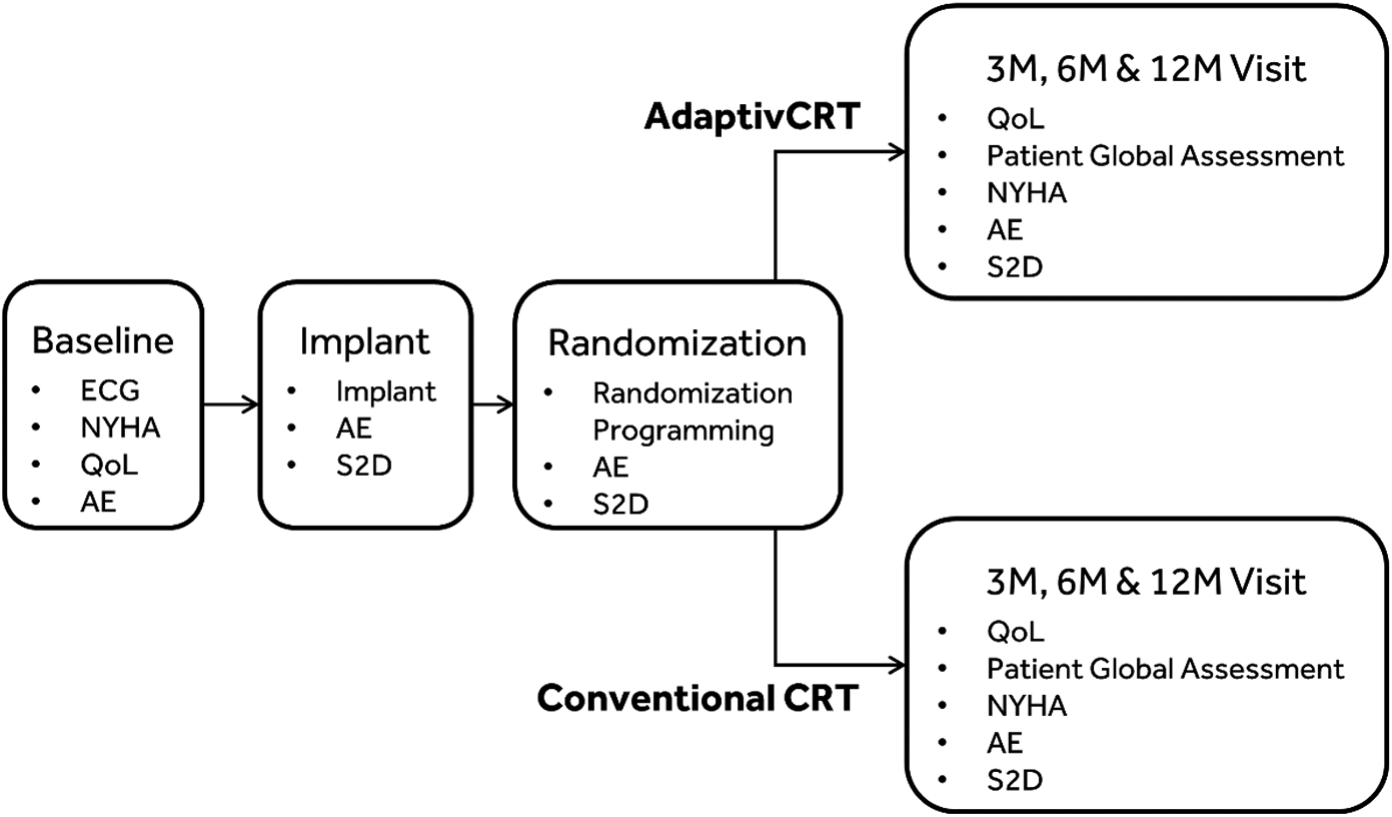
Study flow from baseline to planned study visits. AE, adverse events; CRT, cardiac resynchronization therapy; ECG, electrocardiogram; M, month; NYHA, New York Heart Association; QoL, quality of life; S2D, device data

The flow of enrollment, implant, randomization, and planned follow‐ups is shown in Figure 1. The baseline visit must occur within 14 days after patient enrollment and can be a stand‐alone visit or can be performed on the same day of, but prior to the implant procedure. It is strongly recommended to perform the implant after enrollment; however, enrollments are allowed to occur within 3 days after implant. When a patient is enrolled after implant, the baseline data should reflect the patient status pre‐implant and the baseline visit must be completed within 5 days after implant. After a successful implant, the patient is randomized 1:1 to AdaptivCRT ON vs OFF and the CRT device is programmed accordingly. Following randomization, study patients will be followed at 3, 6, and 12 months, after which study participation is complete and the patient is exited from the trial. Adverse events, changes in cardiovascular medications, echocardiogram data, Patient Global Assessment (PGA), NYHA class, and HF symptoms will be collected. Quality of Life and health outcome will be addressed using the Kansas City Cardiomyopathy Questionnaire (KCCQ) questionnaire, which patients will complete during applicable study visits. Full device interrogation data will be collected for patients at the end of the visit.

### 2.4 Crossover

Every reasonable effort will be made to keep patients in their blinded randomization assignment for the duration of the study. Unless required by clinical or technical urgency, the reprogramming of AdaptivCRT therapy in any patient must be approved by a member of the Steering Committee. Patients will be analyzed per their randomly assigned treatment in accordance with the intent‐to‐treat principle.

### 2.5 Primary objective

The primary objective of the Mid‐Q Response study is to test the hypothesis that AdaptivCRT increases the proportion of patients that Improve on the CCS compared to conventional BiV pacing at 6 months of follow‐up.

**FIGURE 2:**
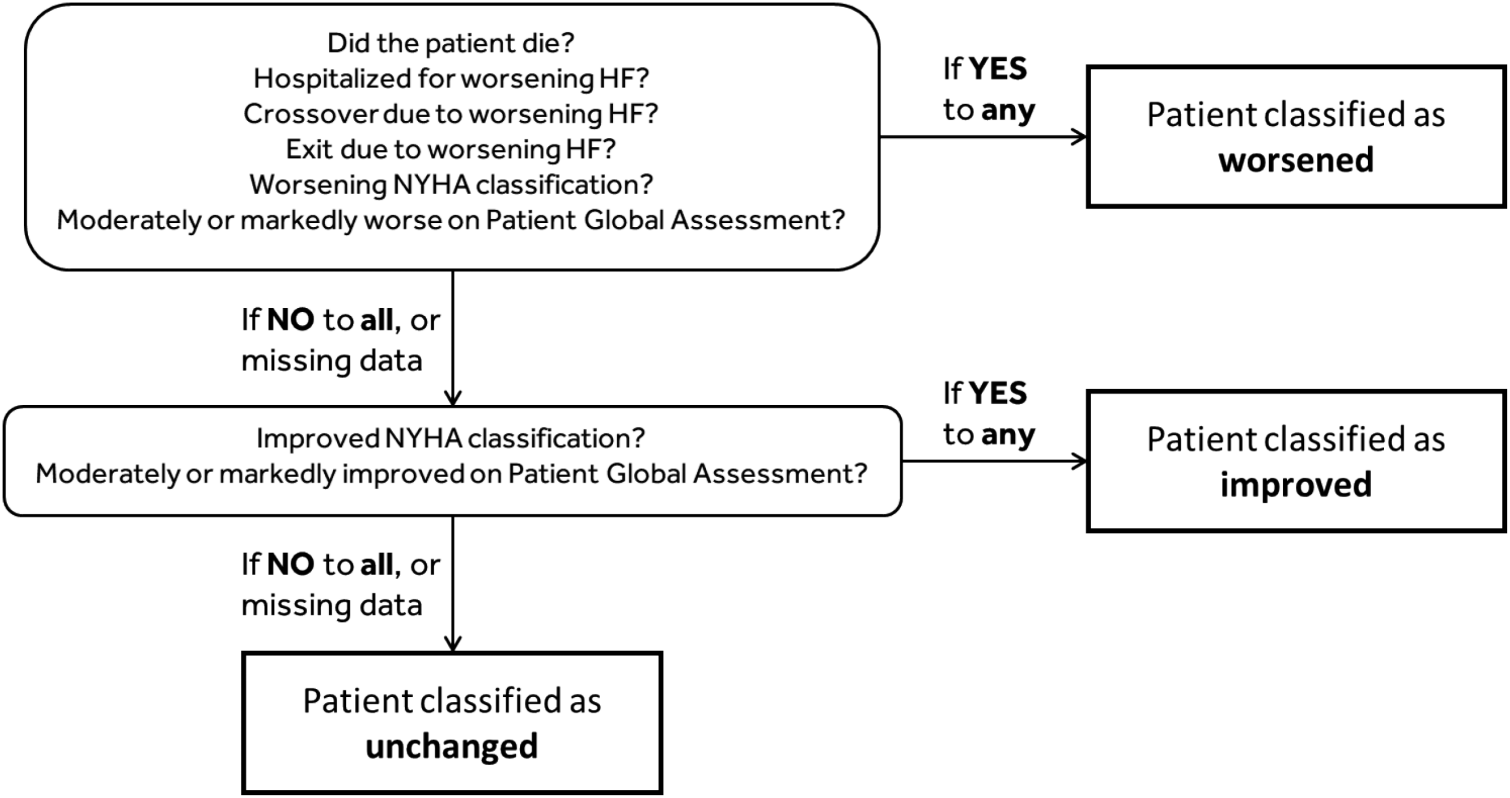
Clinical composite score definition. HF, heart failure; NYHA, New York Heart Association

The CCS classifies patients according to their clinical status at 6 months post‐randomization into categories of Improved, Unchanged, and Worsened as shown in Figure 2. A patient is classified as Worsened in the event of death, hospitalization for worsening HF, worsened NYHA class or worsened status on the PGA. Also, patients that exit the study or cross over because of worsening HF are classified Worsened. A patient is classified as Improved when not Worsened and there is an improvement in NYHA class or PGA. Patients that are not Worsened or Improved are classified as Unchanged, including all patients that miss NYHA class and PGA data who are not classified as Worsened. The main analysis will examine the percentage of patients with Improved CCS. The analysis will include all randomized patients and will follow the intent‐to‐treat principle. A sensitivity analysis will be done including only the patients for whom the ECG Core Laboratory confirmed the presence of LBBB.

### 2.6 Secondary and ancillary endpoints

Prespecified secondary endpoints are all‐cause and cardiovascular‐related mortality, hospitalizations for worsening HF, and NYHA class at 6 and 12 months. Ancillary endpoints are the KCCQ at 6 and 12 months, the incidence of AF, reverse remodeling as measured via echocardiography (LVEF and LV end‐systolic volume), and cardiovascular adverse events. A further ancillary objective is to assess the combined effects of height, QRS duration, and AdaptivCRT on clinical outcomes, as measured by the CCS.

### 2.7 Sample size assumptions

Assuming CCS is improved in 75% of patients in the AdaptivCRT arm and 55% of patients in the conventional BiV arm, a minimum of 220 patients must be randomized to achieve a power of 80% to demonstrate a significant difference in the percentage of patients with Improved CCS. This assumes between‐arm crossover is 3%. Pre‐randomization attrition is expected to be ≤5%, so a total enrollment of 232 patients will meet the sample size requirements.

### 2.8 Statistical analysis

For the primary objective, the percentage of patients with an Improved CCS will be compared using a Chi‐square test. A *p*‐value <0.05 will be considered significant. A sensitivity analysis will be done using a logistic regression model with randomization arm, gender, and NYHA class as fixed effects, and site as a random effect.

Statistical methods used for the analysis of secondary and ancillary objectives will include mixed‐effects logistic regression, ordinal proportional odds logistic regression, and ordinary and competing‐risk survival analysis methods. Subgroups may be considered, including age, gender, body height, HF etiology, LVEF, NYHA class, and QRS duration.

## 3 DISCUSSION

CRT is an established therapy for patients with HF symptoms, LV systolic dysfunction, and a wide QRS complex^1^, ^2^; however, the clinical and hemodynamic benefits of CRT vary significantly among its recipients with limited clinical improvement in up to one third.^3^ Although patient factors may contribute to the response, device factors also play a role. Therefore, optimization of CRT therapy via device settings is an important consideration.

The AdaptivCRT algorithm has been developed to provide RV‐synchronized LV pacing when intrinsic AV conduction is normal or BiV pacing otherwise. The algorithm also adjusts AV and VV delays based on the periodic automatic evaluation of intrinsic conduction intervals. The Adaptive CRT pre‐market approval study demonstrated that AdaptivCRT‐optimized CRT is at least as effective as echo‐optimized BiV pacing in terms of CCS (73.6% improved in the AdaptivCRT arm vs. 72.5% in the echo optimized arm, with a non‐inferiority margin of 12%, *p* = 0.0007).^10^ A post hoc subgroup analysis showed that in patients with sinus rhythm, normal AV conduction, and LBBB, more AdaptivCRT patients improved in their CCS compared with the echo‐optimized arm (80.7% vs. 68.4%, *p* = 0.04). The AdaptivCRT patients in this subgroup received LV‐only pacing 64.0% ± 32.8% of the time.^10^ An additional post hoc analysis of the Adaptive CRT study focused on the patients with normal AV conduction, LBBB, and moderately wide QRS duration (120–150 ms) and also found a greater proportion of patients with an improved CCS in the AdaptivCRT arm than in the echo arm (79% vs. 50%).^11^ In addition, a single‐center, retrospective study of Japanese patients demonstrated that the AdaptivCRT algorithm reduced the risk of the composite of cardiac death or HF hospitalization (hazard ratio: 0.12, 95% CI: 0.006–0.69, *p* = 0.015) in patients with mildly wide QRS, also defined as QRS duration ≥120 ms and <150 ms.^14^

Further investigation of clinical outcomes over longer follow‐up is needed to strengthen the evidence of the benefit of AdaptivCRT. Therefore, the ongoing AdaptResponse study was designed to test the hypothesis that the AdaptivCRT algorithm reduces the incidence of total mortality and HF decompensation events, increases the proportion of patients with an improved CCS, and reduces the incidence of AF in CRT patients with normal AV conduction and LBBB.^15^ The inclusion criteria of AdaptResponse are similar to this Mid‐Q Response study with the exception of QRS duration, which in AdaptResponse is ≥130 ms for women and ≥140 ms for men.^13^ In the past, a wide QRS was typically defined as a QRS duration ≥120 ms. However, in recent years, new clinical evidence raised doubts about the benefit of CRT in women with a QRS duration <130 ms and men with a QRS duration <140 ms. This has led to the implementation of stricter QRS criteria in ACCF/AHA/HRS guidelines and ESC guidelines for CRT implantation.^1^, ^2^

The U.S. and European guidelines do not consider ethnic differences, which have been observed in QRS duration, in the recommendation of CRT implantation. In the general population, Asian adults were found to have narrower QRS complexes compared to Caucasian adults, possibly related to smaller heart sizes in Asians. In addition, the association between LVEF and QRS duration has been observed between Caucasian and Asian patients with HF.^12^ Asian HF patients had shorter QRS durations, smaller body sizes, and more severely impaired LVEF. In the Japanese CRT guidelines, the QRS duration for CRT indication for patients with drug‐resistant HF, LBBB, sinus rhythm, LVEF ≤35%, and NYHA III /IV is set to ≥120 ms.^16^ In other Asian countries, it is also permissible to implant a CRT device in HF patients with a QRS duration of 120 ms or greater.

A recent individual‐patient data meta‐analysis of five randomized controlled trials by Linde et al. looked at the association of sex, QRS duration, and patient height with the benefit of CRT.^17^ For all‐cause mortality, QRS duration was the only independent predictor of CRT benefit. For the composite of all‐cause mortality or first hospitalization for HF, height and QRS duration, but not sex, were independent predictors of CRT benefit. Further analysis suggested that among men with QRS <150 ms, shorter patients had a greater benefit and that smaller men may still have a benefit from CRT even with QRS durations below 140 ms. In a retrospective analysis, 510 patients from the Japanese multicenter CRT database were divided in five subgroups: LBBB and QRS ≥150 ms (*n* = 200, 39%); LBBB and QRS 120–149 ms (*n* = 60, 16%); non‐LBBB with QRS ≥150 ms (*n* = 61, 12%); non‐LBBB with QRS 120–149 ms (*n* = 54, 11%), and narrow QRS complex with QRS <120 ms (*n* = 115, 23%) in order to study CRT outcomes.^18^ Echocardiographic response, defined as a relative reduction of left ventricular end‐systolic volume (LVESV) ≥15% at 6–12 months after CRT implantation, in the subgroups was 74%, 51%, 38%, 52%, and 50% respectively, *p* < 0.001. The incidence of the primary endpoint (composite of all‐cause death or hospitalization because of HF) was significantly different between the groups as well, even after adjusting for other baseline characteristics (28.6%, 42.3%, 45.9%, 55.6%, and 55.3% respectively, *p* < 0.001).

From the above studies, it was collectively hypothesized that patients in Asia can benefit from CRT in general and AdaptivCRT in particular, even at the lower end of the QRS duration spectrum because the population is smaller in height compared to the average patient population in the landmark CRT studies, and because AdaptivCRT is more effective than standard BiV pacing in the subgroup of patients with a mid‐range QRS of 120–149 ms. A moderately widened QRS in smaller Asian patients may reflect the presence of electromechanical dyssynchrony of a similar degree to that observed at much wider QRS in larger Caucasian patients. Showing the benefit of AdaptivCRT therapy in the moderately wide QRS cohort through this study may help to give all HF patients who would benefit from CRT access to this therapy.

## 4 CONCLUSION

The Mid‐Q response study aims to demonstrate the incremental benefits of the AdaptivCRT algorithm compared to conventional BiV pacing on clinical outcomes in Asian patients with a moderate QRS duration, LBBB, and normal AV conduction.

## CONFLICT OF INTEREST

Kengo Kusano, MD, PhD: Speaker honoraria from Daiichi Sankyo Company, Ltd., Japan, Bristol‐Myers Squibb, Biotronik Japan, and Medronic Japan, and research grants from Medtronic Japan. Seung‐Jung Park, MD, PhD: Research grants and speaker honoraria from Boston Scientific Korea, Abbott Korea, Biotronik Korea, and Medtronic Korea. Sofian Johar, MB, BChir, PhD: speaker for Medtronic and Johnson and Johnson. Toon Wei Lim MD, PhD: Speaker for Biotronik, Medtronic, and Abbott. Research support from Medtronic and Biotronik. Kazuhiro Hidaka: employee and shareholder of Medtronic. Bart Gerritse: employee and shareholder of Medtronic. Kazutaka Aonuma, MD, PhD: Speaker honoraria from Boehringer Ingelheim GmbH, Medtronic Japan Co., Ltd., Daiichi Sankyo Co., Ltd., Japan, and Abbott Medical Japan Co., Ltd., and research grants from Otsuka Pharmaceutical Co., Ltd, ASTEC Co., Ltd, Astellas Pharma Inc., Johnson & Johnson, Takeda Pharmaceutical Co., Ltd, TEIJIN PHARMA LIMITED, SHIONOGI & CO., LTD., Medical Corporation Tsukuba Kinenkai, Abbott Medical Japan Co., Ltd., and belongs to endowed departments by Boston Scientific Corporation, Japan Lifeline Co., Ltd., Nihon Cohden Corporation, BIOTRONIK Japan Inc., Toray Industries, Inc., Boehringer Ingelheim GmbH, Century Medical Inc.

## APPENDIX A

### A.1

**MidQ Steering Committee**

Kazutaka Aonuma, MD, Chair, Tsukuba University, Tsukuba, Japan; Kengo Kusano, MD, National Cerebral and Cardiovascular Center, Osaka, Japan; Seung Jung Park, MD, Samsung Medical Center, Seoul, South Korea; Sofian Johar, MD, Glemeagles Jerudong Park Medical Centre and Institute of Health Sciences, Universiti Brunei Darussalam, Brunei Darussalam; and Lim Toon Wei, MD, National University Hospital, Singapore.
